# HaploCatcher: An R package for prediction of haplotypes

**DOI:** 10.1002/tpg2.20412

**Published:** 2023-11-15

**Authors:** Zachary James Winn, Emily Hudson‐Arns, Mikayla Hammers, Noah DeWitt, Jeanette Lyerly, Guihua Bai, Paul St. Amand, Punya Nachappa, Scott Haley, Richard Esten Mason

**Affiliations:** ^1^ Department of Soil and Crop Sciences Colorado State University Fort Collins Colorado USA; ^2^ School of Plant, Environmental, and Soil Sciences Louisiana State University Baton Rouge Louisiana USA; ^3^ Department of Crop and Soil Sciences North Carolina State University Raleigh North Carolina USA; ^4^ USDA Agricultural Research Service Hard Winter Wheat Genetics Research Unit Manhattan Kansas USA; ^5^ Department of Agricultural Biology Colorado State University Fort Collins Colorado USA

## Abstract

Wheat (*Triticum aestivum* L.) is crucial to global food security but is often threatened by diseases, pests, and environmental stresses. Wheat‐stem sawfly (*Cephus cinctus* Norton) poses a major threat to food security in the United States, and solid‐stem varieties, which carry the stem‐solidness locus (*Sst1*), are the main source of genetic resistance against sawfly. Marker‐assisted selection uses molecular markers to identify lines possessing beneficial haplotypes, like that of the *Sst1* locus. In this study, an R package titled “HaploCatcher” was developed to predict specific haplotypes of interest in genome‐wide genotyped lines. A training population of 1056 lines genotyped for the *Sst1* locus, known to confer stem solidness, and genome‐wide markers was curated to make predictions of the *Sst1* haplotypes for 292 lines from the Colorado State University wheat breeding program. Predicted *Sst1* haplotypes were compared to marker‐derived haplotypes. Our results indicated that the training set was substantially predictive, with kappa scores of 0.83 for *k*‐nearest neighbors and 0.88 for random forest models. Forward validation on newly developed breeding lines demonstrated that a random forest model, trained on the total available training data, had comparable accuracy between forward and cross‐validation. Estimated group means of lines classified by haplotypes from PCR‐derived markers and predictive modeling did not significantly differ. The HaploCatcher package is freely available and may be utilized by breeding programs, using their own training populations, to predict haplotypes for whole‐genome sequenced early generation material.

## Core Ideas

- Identification, introgression, and frequency increase of large effect loci are important for cultivar development.
- The *Sst1* locus has a significant effect on cutting score in fields exposed to sawfly infestation.
- Historical genetic information can be utilized to predict haplotypes for lines which have genome‐wide genetic data.
- An R package, HaploCatcher, has been developed to facilitate this analysis in other programs.

**Abbreviations**
AYN: advanced yield nursery
BLUE: best linear unbiased estimate
IWGSC: International Wheat Genome Sequencing Consortium
KASP: kompetative allele‐specific polymerase chain reaction
KNN: k‐nearest neighbors
LD: linkage disequilibrium
Mbp: megabase pair
PCR: polymerase chain reaction
QTL: quantitative trait loci
RF: random forest
RGON: Regional Germplasm Observation Nursery
SNP: single nucleotide polymorphism
SRPN: Southern Regional Performance Nursery
USDA: United States Department of Agriculture
WSS: wheat stem sawfly solid stem panel

## 1 INTRODUCTION

Common bread wheat (*Triticum aestivum* L.) consumption represents nearly 20% of human caloric intake; however, current genetic gain of wheat grain yield is insufficient to meet the rise in demand as the global population increases (Poole et al., 2021; Ray et al., 2013; Shiferaw et al., 2013). Two major threats to grain yield stability in wheat are diseases and pests. One such pest, which presents a major risk to yield stability in the US northern Great Plains and Mountain West regions, is wheat stem sawfly (*Cephus cinctus* Norton). In terms of domestic losses, the Colorado winter wheat growing region lost ∼32.7 and 31.2 million dollars’ worth of wheat in the years 2020 and 2021, respectfully (Erika et al., 2023). Yield losses for infested hollow‐stem varieties can be anywhere from 90 to 120 kg ha^−1^ for spring wheats, potentially resulting in multi‐million dollar losses annually (Beres et al., 2007). Furthermore, Beres et al. (2011) estimated that sawfly infestation may cost 350 million dollars annually to the US northern Great Plains and Canadian provinces, making it a major concern of consumers and producers alike.

The wheat stem sawfly is an insect native to North America that infests wheat by ovipositing eggs within the stem of wheat plants from late May to early June (Weiss & Morrill, 1992). Once the egg has been deposited into the stem, the larva emerges and feeds upon the parenchyma and vascular tissue inside the stem (Weiss & Morrill, 1992). After receiving the correct combination of physical and photoperiodic signals (Holmes, 1975), the larvae will migrate downward from its hatching site to an area of the stem near the soil surface and create a notch, which is known as a hibernaculum, that it fills with the excrement of digested plant material (frass) (Weiss & Morrill, 1992). The wheat stem tends to break at the notch development site, causing affected plants to lodge, which results in substantial yield losses. After the establishment of the hibernaculum, the larvae will enter a period of diapause to then pupate and emerge from the hibernaculum in the following year (Beres et al., 2011).

Although wheat stem sawflies are weak fliers that tend to oviposit near the area of emergence (Beres et al., 2011; Weiss & Morrill, 1992), their distribution is wide, and integrated pest management is challenging. Removal of stubs was once recommended as a key cultural control of wheat stem sawfly (Fletcher, 1904), but contemporary research has demonstrated that it is an ineffective control method (Beres et al., 2011). Rotations of no‐till wheat followed by fallow also appear to increase infestation rates, and this phenomenon is mainly attributed to the reduction of tillage in these rotations, which allows emerging adult sawfly to easily migrate from stubble in strips of fallow land to wheat plots planted in adjacent fields (Beres et al., 2011; Seamens, 1929).

In lieu of leaving land fallow, it has been suggested to use a nonhost crop in rotation as a “trap crop.” More recently, trap crops have been suggested as a management tool where a nonhost plant is planted as a border around hollow‐stem varieties to act as a buffer zone and prevent infestation of higher yielding hollow‐stem varieties (Beres et al., 2009; Peirce, Cockrell, Ode, et al., 2022). Regardless of these management tools, genetic resistance remains the most effective management practice.

**Core Ideas**

- Identification, introgression, and frequency increase of large effect loci are important for cultivar development.
- The *Sst1* locus has a significant effect on cutting score in fields exposed to sawfly infestation.
- Historical genetic information can be utilized to predict haplotypes for lines which have genome‐wide genetic data.
- An R package, HaploCatcher, has been developed to facilitate this analysis in other programs.

Solid‐stem varieties of wheat have been available since the mid‐20th century (Peirce, Cockrell, Mason, et al., 2022; Weiss & Morrill, 1992). In solid‐stem varieties, undifferentiated parenchyma cells create a solid pith within the stem (Berzonsky et al., 2003). Due to the partitioning of biomass to the stem, it has been hypothesized that the yield potential of lines with the solid‐stem locus may be limited in comparison to hollow‐stem varieties, which has made the adoption of solid‐stem varieties by growers slow (Weiss & Morrill, 1992). However, lines that express stem‐solidness have less yield losses in comparison to hallow‐stem wheat plants in the presence of sawfly pressure (Beres et al., 2007), and this has prompted the adoption of solid‐stem varieties by growers who have annual wheat stem sawfly infestation.

The genetic architecture of stem solidness appears oligogenic, with large effect loci being the main contributors to solidness (Peirce, Cockrell, Mason, et al., 2022). One gene found responsible for solidness is *Sst1* (Nilsen et al., 2017, 2020), which was first identified in a quantitative trait locus (QTL) mapping  study conducted by Cook et al. (2004) on the long arm of wheat chromosome 3B (*Qss.msub‐3BL*). Stem solidness is thus caused, in part, by tandem repeats of the *TdDof* gene coding sequence which leads to the filling of the pith within the wheat stem (Nilsen et al., 2020).

While the visual rating of stem solidness can be a reliable method for selecting lines that express solid‐stem phenotypes, many wheat breeders in the United States utilize molecular markers to haplotype the region containing *Sst1* in a process termed marker‐assisted selection. More recently, the US Department of Agriculture (USDA) Central Small Grains Genotyping Lab located in Manhattan, Kansas, has been producing haplotype information on many large effect loci, including *Sst1*, for the lines entered into the Southern Regional Performance Nursery (SRPN) and Regional Germplasm Observation Nursery (RGON) (https://www.ars.usda.gov/plains‐area/lincoln‐ne/wheat‐sorghum‐and‐forage‐research/docs/hard‐winter‐wheat‐regional‐nursery‐program/research/). This service performed by the USDA laboratory is conducted to assist breeders in releasing lines with the solid‐stem trait, and as a result, it has also created a backlog of information on lines in the SRPN and RGON lines with known *Sst1* haplotypes. Moreover, the lines in the SRPN and RGON have been characterized for genome‐wide single nucleotide polymorphisms (SNPs) by the Colorado State University Wheat Breeding Program on an annual basis for more than a decade.

Winn et al. (2022) described a method where historical molecular and haplotype data are utilized to produce accurate haplotype predictions for lines which only have genome‐wide molecular data. In brief summary, Winn et al. (2022) trained three machine learning algorithms (k‐nearest neighbors, random forest, and gradient boosting machine) on marker‐assay‐based haplotypes for four loci (*Fhb1*, *QFhb.nc‐1A*, *QFhb.nc‐4A*, and *QFhb.vt‐1B*) related to *Fusarium* head blight (*Fusarium graminearum* Schwabe) resistance. Using those trained models, Winn et al. (2022) demonstrated that historical haplotype information could be used in conjunction with genome‐wide molecular marker data to produce accurate predictions of QTL haplotypes. However, the methods proposed by Winn et al. (2022) were developed using *Fusarium* head blight resistance loci in soft red winter wheat germplasm and have yet to be applied in unrelated populations of wheat for different loci.

In the current study, we sought to (1) produce a deployable R statistical software compatible package to perform an analysis similar to the one performed in Winn et al. (2022), (2) demonstrate that the analysis preforms similarly in an unrelated germplasm pool for a different locus than those explored in Winn et al. (2022), (3) predict the *Sst1* haplotypes of genome‐wide genotyped individuals, and (4) compare the estimated means of predicted *Sst1* versus genotyped *Sst1* haplotypes using stem sawfly‐related phenotypes in Colorado State University hard winter wheat germplasm.

## 2 MATERIALS AND METHODS

### 2.1 Germplasm

Two separate sets of germplasm were utilized in this study. The first population used in this study was a historical panel of lines submitted to the SRPN and RGON. This panel of lines consisted of 1056 distinct genotypes, and all lines in the panel were genotyped for genome‐wide SNPs and haplotyped via a diverse panel of markers for the *Sst1*/*Qss.msub‐3BL* locus. The second population utilized in this study represents contemporary lines in the Colorado State University Wheat Breeding Program from the 2022 advanced yield nursery (AYN) and the 2022 wheat stem sawfly solid stem panel (WSS). Lines in the 2022 AYN and WSS were phenotyped for sawfly reaction traits, genotyped for SNPs across the genome, and screened with kompetative allele‐specific polymerase chain reaction (KASP) assays for the *Sst1* locus. The AYN consisted of 107 distinct genotypes, and the WSS consisted of 185 distinct genotypes (292 total genotypes). Individuals in the WSS had not gone through any phenotypic or marker‐assisted selection for solid stem or wheat stem sawfly resistance, while individuals in the AYN had already undergone one generation of phenotypic selection for resistance.

### 2.2 Phenotyping

In the 2021–2022 wheat growing season, the AYN and WSS were planted in Akron, Colorado, and a second location of the AYN was planted in New Raymer, Colorado. These sites were selected for sawfly trials due to the historical presence of sawfly within these regions and the consistent infestation that they receive (Cockrell et al., 2021; Irell & Peairs, 2014). Furthermore, scouting of areas in proximity to field sites was performed within the 2021–2022 wheat growing season by sweeping boardering plots with entomology nets 100 times. Sampling began during mid‐jointing and continued weekly until no adult sawfly was found in sweep samples. These data further confirmed that infestation pressure was adequate for data collection within selected field sites (P. Nachappa, personal communication, 2023; Nachappa & Peirce, 2022).

In each site, all plots were sown in mid‐September using a 1.5‐m wide no‐till drill seeder that was guided by a cable and had 4.9‐m spacing between centers. Following spring green up, centers were pruned using glyphosate (Bayer) applied by a 1.2‐m wide hooded sprayer. After end trimming, this resulted in a measurable area of 1.5 m by 3.7 m. The AYN and WSS were planted in partially replicated designs arranged in rows and columns, with repeated checks included at both row and column levels.

After physiological maturity, when lodging due to sawfly cutting was apparent, a visual cutting score was assigned to each plot in each location. The visual cutting score was assigned as an index of percent plot affected by cutting, which is the physical process by which insect injury detaches most of the wheat stem from the base of the plant. Visual scores were assigned via an ordinal scale ranging from 1 to 9, where 1 is fully resistant and erect despite wheat stem sawfly pressure, and 9 indicates the whole plot is affected, cut, and prostrate.

### 2.3 Genome‐wide genotyping

Ten seeds were planted for each line, and a 2‐ to 3‐cm leaf tissue sample was taken from each plant and bulked for DNA extraction. Genomic DNA was extracted from the samples using MagMax (ThermoFisher Scientific) plant DNA kits following the manufacturer's instructions and quantified using PicoGreen (ThermoFisher Scientific) kits. Extracted DNA was normalized to a concentration of 20 ng µL^−1^, and sequencing libraries were prepared following the protocol established by Poland et al. (2012). The multiplexed libraries were sequenced on a NovaSeq 6000 (Illumina) sequencer at 384‐plex density per lane. The resulting reads were aligned to the International Wheat Genome Sequencing Consortium (IWGSC) wheat reference sequence RefSeq v2.0 (Appels et al., 2018) using the burrow‐wheeler aligner (Li & Durbin, 2009).

The TASSEL 2.0 standalone pipeline (Glaubitz et al., 2014) version 5.0 was used to process the reads obtained from alignment, and markers were organized into compressed variant calling format files (Danecek et al., 2011). Initial variant calling format files were filtered using the following parameters: monomorphic SNPs, insertions, and deletions were removed; SNPs with 85% or less missing data were retained; SNPs with a read depth of more than one or less than 100 were retained; SNPs with a minimum allele frequency of less than 5% were removed; SNPs with more than 10% heterozygosity were removed; and all unaligned SNPs were removed. After filtration, missing data were imputed using the Beagle algorithm V5.4 (Browning et al., 2018), and a synthetic wheat biparental cross between “W7984” and “Opata” was used to derive a recombination distance‐based map for imputation (Gutierrez‐Gonzalez et al., 2019).

### 2.4 Historical haplotype information curation

Information on the *Sst1* locus was curated from historical marker calling files generated by the USDA Central Small Grains Genotyping Lab (https://www.ars.usda.gov/plains‐area/lincoln‐ne/wheat‐sorghum‐and‐forage‐research/docs/hard‐winter‐wheat‐regional‐nursery‐program/research/). The information for lines in both the RGON and SRPN was standardized to a biallelic haplotype of homozygous *Sst1*, heterozygous, and homozygous wild‐type represented as “+/+,” “+/−,” and “−/−,” respectively.

Haplotype calls within the CSU wheat breeding program were made using a single marker identified as diagnostic for the *Sst1* locus. Extracted and purified DNA, ranging between 50 and 150 ng µL^−1^, were plated in 96‐well plates in 4 µL volumes. Plates contained both test genotype DNA as well as positive, heterozygous, negative, and non‐template controls. Each well contained 4 µL of 2X KASP (LGC Genomics) reaction mixture and 0.11 µL of KASP primer assay mixture. The assay mixture contained an equal mixture of 100 µM of FAM and HEX fluorescence‐labeled forward primers, as well as a 2.5 concentration of 100 µM reverse primer, suspended in molecular‐grade sterile water (Table 1). Assays were run on a Bio‐Rad (Bio‐Rad) CFX96 RT PCR machine, and results were read using a single endpoint measurement of florescence. Haplotype calls were made by visual discrimination of florescence groupings. The frequency of allelic states for *Sst1* in the training and testing set is also provided (Table 2).

**TABLE 1:** List of primers and sequences for the *Usw275* marker.

Marker	Chromosome	Position (Mbp^a^)	Primer	Sequence
*Usw275*	3B	843.6	HEX forward^b^	GAAGGTCGGAGTCAACGGATTAAAGAAAACAAAACCTGTCAAAAAC
			FAM forward^c^	GAAGGTGACCAAGTTCATGCTAAAGAAAACAAAACCTGTCAAAAAT
			Common reverse	GAATTTTCGGAGTTACAGATTGC

^a^ Megabase pair position.

^b^ HEX labeled primer is diagnostic for the solid allele of the *Sst1* locus.

^c^ FAM labeled primer is diagnostic for the nonsolid allele of the *Sst1* locus.

**TABLE 2:** Number of observations and frequency of *Sst1* haplotypes in the training and test population.

	Training		Testing	
Haplotype	*n* ^a^	Frequency	*n*	Frequency
+/+^b^	303	0.29	156	0.53
+/−^c^	104	0.10	25	0.09
−/−^d^	649	0.61	111	0.38

^a^ Number of observations.

^b^ Homozygous *Sst1* haplotype.

^c^ Heterozygous *Sst1* haplotype.

^d^ Homozygous wild‐type haplotype.

### 2.5 Package development and analysis pipeline

**FIGURE 1:**
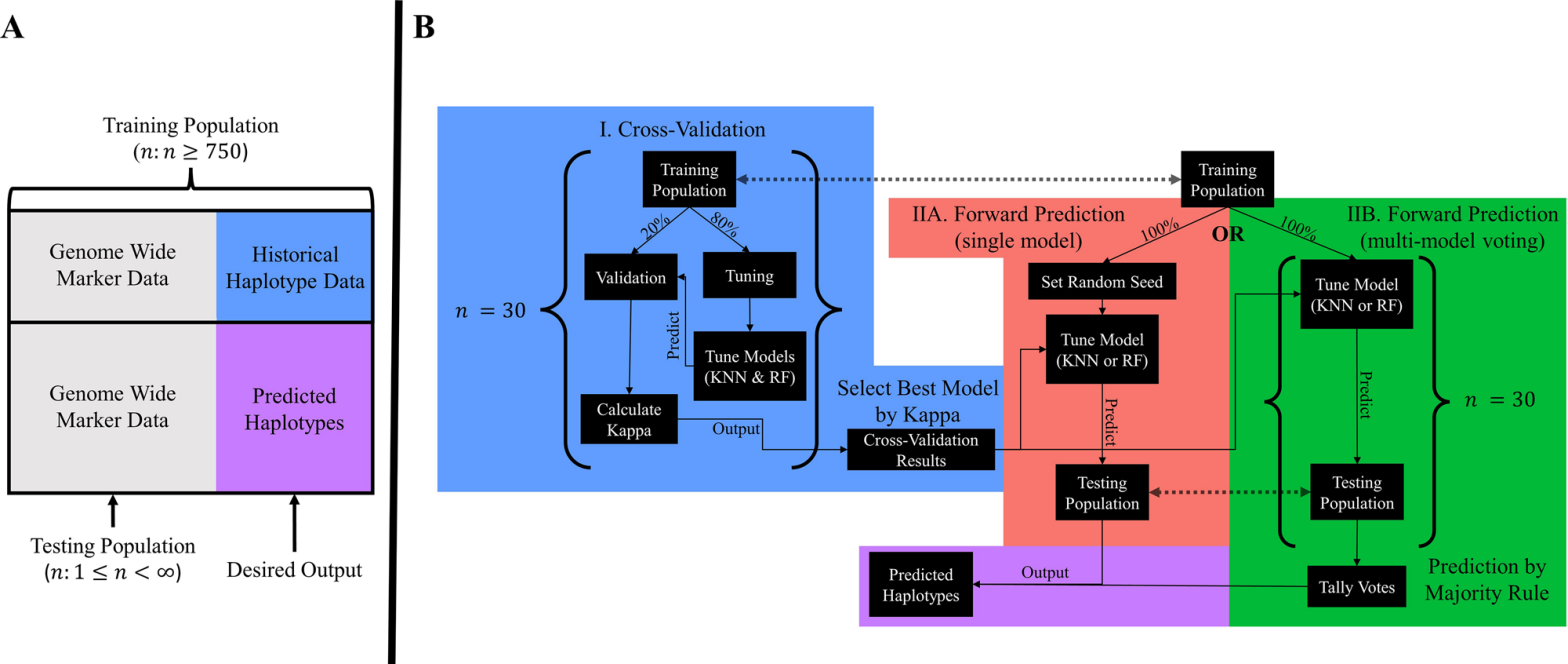
A diagram of (A) input data structure and (B) the “auto_locus” function pipeline. Panel (A) shows a total dataset that is partitioned into a training and test population. The training population in panel (A) shows a population of individuals, that is suggested to be composed of more than 750 individuals, which have both genome‐wide marker and historical haplotype data. The testing population in panel (A) shows a testing population, which may be any size greater than zero, which only has genome‐wide marker data. Panel (B) shows the workflow of the “auto_locus” function. In the cross‐validation step (I), the total training population is split in a user‐defined way (default is 80:20 split) and the 80% tuning population is used to train and select optimal hyper‐parameters for a k‐nearest neighbors (KNN) and random forest (RF) model. The trained models are then used to predict the haplotype of the validation population. The predicted haplotype is then compared to the "true" haplotype, and kappa (and accuracy) is calculated. This is repeated a user‐set number of times (default is 30). The best performing model based on accuracy or kappa (default is kappa) is then taken as the model to be used in forward prediction. There are two options post cross‐validation: (IIA) a single model with a set seed for repeatability or (IIB) a user‐set number of random models (default is 30) used to create a consensus haplotype prediction.

The “HaploCatcher” package was developed using the “devtools” package (Wickham et al., 2022) in R statistical software (R Core Team, 2022) via the RStudio (Posit) development environment on a computer with a Microsoft Windows operating system. The package is composed of several core functions which are then streamlined into the function “auto_locus.” The “auto_locus” function conducts a similar analysis pipeline to Winn et al. (2022) through the “caret” package (Kuhn, 2008, 2022), while requiring minimal intervention from users (Figure 1b).

There are three main inputs required for the “auto_locus” function: a genotypic matrix, a marker information file, and a “gene compendium.” The first input provided by the user is a genotypic matrix that contains an imputed, number‐coded genotype matrix which has dimensions of *n* number of individuals and *m* number of markers where the row names are genotypes, and the column names are markers. The second input provided by the user is the marker information file that contains the name, chromosome (or linkage group), and position (either base pair or centimorgan) of every marker found in the genotype matrix file.

The third input provided by the user is a “gene compendium” that contains at least four columns titled “FullSampleName,” “Gene,” “Chromosome,” and “Call,” which are case sensitive. The “FullSampleName” column is the names of the genotypes that relate to the row names of the genotypic matrix; these names are case sensitive and must be identical. The “Gene” column has the name of the gene. The “Chromosome” column contains the name of the chromosome (or linkage group) where the gene is found; this chromosome name is case sensitive and must match a chromosome identified in the marker information file. The final column, “Call,” contains the haplotype of the specified genotype at that locus. For more information on input files and formatting, users may refer to supplemental codes (Supporting Information).

The “auto_locus” function is composed of two major phases: cross‐validation by random partitioning into a user‐specified ratio over a set number of permutations (Figure 1b—Step I) and forward prediction of candidates by the best model in cross‐validation (Figure 1b—Step IIA and Figure 1b—Step IIB). In cross‐validation, two models are evaluated for the prediction of the training data: k‐nearest neighbors (KNN) and random forest (RF).

In KNN, a user‐set number of nearest neighbors (default is 50 tested in groups of 5 [1, 5, …, 50]) is determined by a user‐defined number of markers that are most correlated (determined by the absolute value of the Pearson's correlation coefficient) to the provided haplotype information (default is 50). In RF, a series of decision trees are made, and randomly split nodes are tested based on the number of correlated markers provided (default is 50 and tested in groups of 5 [1, 5, …, 50]). The number of trees in the random forest is optimized by the “caret” package.

The number of cross‐validation permutations performed is user defined (default is 30), and two metrics can be used to determine the best performing model: categorical accuracy or Cohen's *κ* (default is kappa). Once the best performing model is selected by the user‐defined metric of assessment, the indicated best‐performing model may be used in two methods of forward prediction: either setting a random seed for reproducibility and performing forward prediction once (Figure 1b—Step IIA) or by running the optimal model with no set seed over a user‐specified number of permutations (default is 30; Figure 1b—Step IIB) and producing a haplotype prediction by majority rule. After forward prediction, all lines that are indicated to be a part of the testing population will be assigned a predictive haplotype call, which is accessible in the results object obtained from the “auto_locus” function.

Cross‐validation results are visualized using functions from the packages “ggplot2” and “patchwork” (Pedersen, 2022; Wickham et al., 2016). Both the cross‐validation and forward prediction by voting steps in the “auto_locus” function can be run either sequentially or in parallel using the R packages “parallel,” “doParallel,” and “foreach” (R Core Team, 2022; Microsoft & Weston, 2022a, 2022b). Users can specify if the analysis is to be done in parallel (default argument is FALSE) or sequentially. Users may also define the number of processing cores desired for analysis or use the default setting which uses the function “detectCores” from the parallel package to determine the number of system cores and subtract that value by one.

The computer used for the development of the package had a hexacore 2.6 GHz Intel (Intel) i7‐10750H processor with 12 logical processors and 32 gigabytes of DDR4 RAM. Using the example datasets available in the HaploCatcher package, the “auto_locus” function performed in parallel with 100 permutations of cross‐validations and 100 votes for majority rule resulted in a total runtime of 8 min and 36 s.

### 2.6 Statistical analysis—Mixed linear models

All statistical analyses were conducted in R statistical software version 4.2.2 (R Core Team, 2022). Cutting visual score data were checked for normality by the visualization of the distribution of observations using histograms and QQ‐plots. Upon evaluation, all data exhibited near‐normality or somewhat skewed normal distributions. Mixed linear models were run using the function “mmer” in the package “sommer” (Covarrubias‐Pazaran, 2016, 2018). Across locations, the following model was utilized to estimate the effect of the *Sst1* locus:

(1) $$\;y_{ijkl}=\mu +H_{i}+g_{j}+e_{k}+r:c_{lm}+\varepsilon_{ijklm},$$

where $y_{ijklm}$ is the response, μ is the population mean, $H_{i}$ is the haplotype fixed effect of the *i*th haplotype, $g_{j}$ is the genotypic random effect of the *j*th genotype effect whose variance is defined by the additive relationship matrix among individuals derived from markers (Covarrubias‐Pazaran, 2016; Endelman & Jannink, 2012; VanRaden, 2008), $e_{k}$ is the random environment effect of the *k*th environment that is identically and independently distributed across levels, $r:c_{lm}$ is the random row by column interaction effect of the *l*th row and the *j*th column whose variance is defined by the two‐dimensional penalized tensor‐product of spline relationship between row and column effects as described by Lee et al. (2013), and $\varepsilon_{ijklm}$ is the residual error that is identically and independently distributed across all levels. The additive relationship matrix was computed through a series of steps. First, the marker matrix was centered by subtracting the means of each column. After this, the centered marker matrix was multiplied by the inverse of the marker matrix. Finally, the result of this multiplication was divided by the mean value found on the diagonal of the marker matrix (Covarrubias‐Pazaran, 2016; Endelman & Jannink, 2012).

To compare KASP‐genotyped haplotype versus machine learning‐predicted haplotype group means, the same mixed linear model was run twice to estimate an $H_{i}$ haplotype fixed effect. The first fit of the model was performed using the “true” haplotype calls derived from KASP genotyping, and the second fit of the model was performed using the machine learning‐predicted haplotype information. Fixed effect group mean estimates for both the observed and predicted haplotype means were estimated via the “predict.mmer” function in the package “sommer.” Visual comparison of effect estimates was summarized using functions from the “ggplot2” package.

Narrow‐sense, per‐plot, genomic heritability ($h_{g}^{2}$) of cutting visual score ratings within the environment were estimated using the following mixed linear model:

(2) $$\;y_{i}=\mu +g_{i}+\varepsilon_{i},$$

where $y_{i}$ is the observation, μ is the population mean, $g_{i}$ is the random genotype effect of the *i*th genotype whose variance is defined by the marker‐derived additive relationship matrix calculated by the “A.mat” function from the “sommer” package, and $\varepsilon_{i}$ is the residual error whose variance is identically and independently distributed. Variance components were used in the function “vpredict” in the “sommer” package to estimate $h_{g}^{2}$ using the following formula:

(3) $$\;h_{g}^{2}=\frac{\sigma_{g}^{2}}{\sigma_{g}^{2}+\sigma_{\varepsilon }^{2}},$$

where $h_{g}^{2}$ is the narrow‐sense, per‐plot, genomic heritability; $\sigma_{g}^{2}$ is the genotypic variance; and $\sigma_{\varepsilon }^{2}$ is the residual error variance.

### 2.7 Statistical analysis—Machine learning

The importance of defining variables (genome‐wide SNP markers) in KNN and RF algorithms was calculated for each iteration of the 100 permutations of cross‐validation by using the function “varImp” in the “caret” package. Variable importance, or more aptly put the importance of genome‐wide SNP markers in defining haplotypes, was scaled between zero and 100 for comparability across models, and the average importance of markers across all permutations was reported in images generated by the “ggplot2” package. Linkage disequilibrium (LD) was calculated for all markers identified as important using the function “LD” in the package “gaston,” and results were reported in images derived from functions in the “ggplot2” package (Perdry & Dandine‐Roulland, 2018).

Confusion matrices were calculated by comparing the predicted haplotype to the observed haplotype state in the WSS and AYN combined via the function “confusionMatrix” in the “caret” package. Model performance parameters were calculated across iterations of the 100 permutations of cross‐validation and the forward prediction of the WSS and AYN. The reported measures of model performance were accuracy, sensitivity, specificity, and unadjusted Cohen's *κ* (McHugh, 2012). Accuracy was calculated as:

(4) $$\mathrm{Accuracy}\;=\frac{\mathrm{TP}+\mathrm{TN}}{\mathrm{TP}+\mathrm{TN}+\mathrm{FP}+\mathrm{FN}},$$

where *TP* is the number of true positive cases, TN is the number of true negative cases, FP is the number of false positive cases, and FN is the number of false negative cases. Sensitivity and specificity were calculated as such:

(5) $$\mathrm{Sensitivity}\;=\frac{\mathrm{TP}}{\mathrm{TP}+\mathrm{FN}},$$

(6) $$Specifity\;=\frac{\mathrm{TN}}{\mathrm{TN}+\mathrm{FP}},$$

Cohen's *κ* was calculated as follows:

(7) $$\kappa =\frac{\mathrm{Pr}\left(a\right)-\mathrm{Pr}\left(e\right)}{1-\mathrm{Pr}\left(e\right)},$$

where Pr(*a*) is the probability of observed agreement, and Pr(*e*) represents the expected rate of chance agreement. Kappa is often considered more robust than accuracy as a measurement parameter of reliability in categorization models because it is not easily biased by sample size (McHugh, 2012). Kappa may be understood as a measurement which is bound between −1 and 1 where a value of 1 represents a perfectly categorizing model, 0 is the same as chance agreement, and a value of −1 is categorization that is worse than chance agreement (Viera et al., 2005). Historically, a categorical prediction model which has a kappa value of 0.8 is considered “substantial” in its predictive ability, whereas a model with a kappa of 1 is “perfect’ in its predictive ability (Landis & Koch, 1977). All model parameters were either reported in tables or visualized using functions from the “ggplot2” package.

## 3 RESULTS

### 3.1 *Sst1* prediction cross‐validation

**FIGURE 2:**
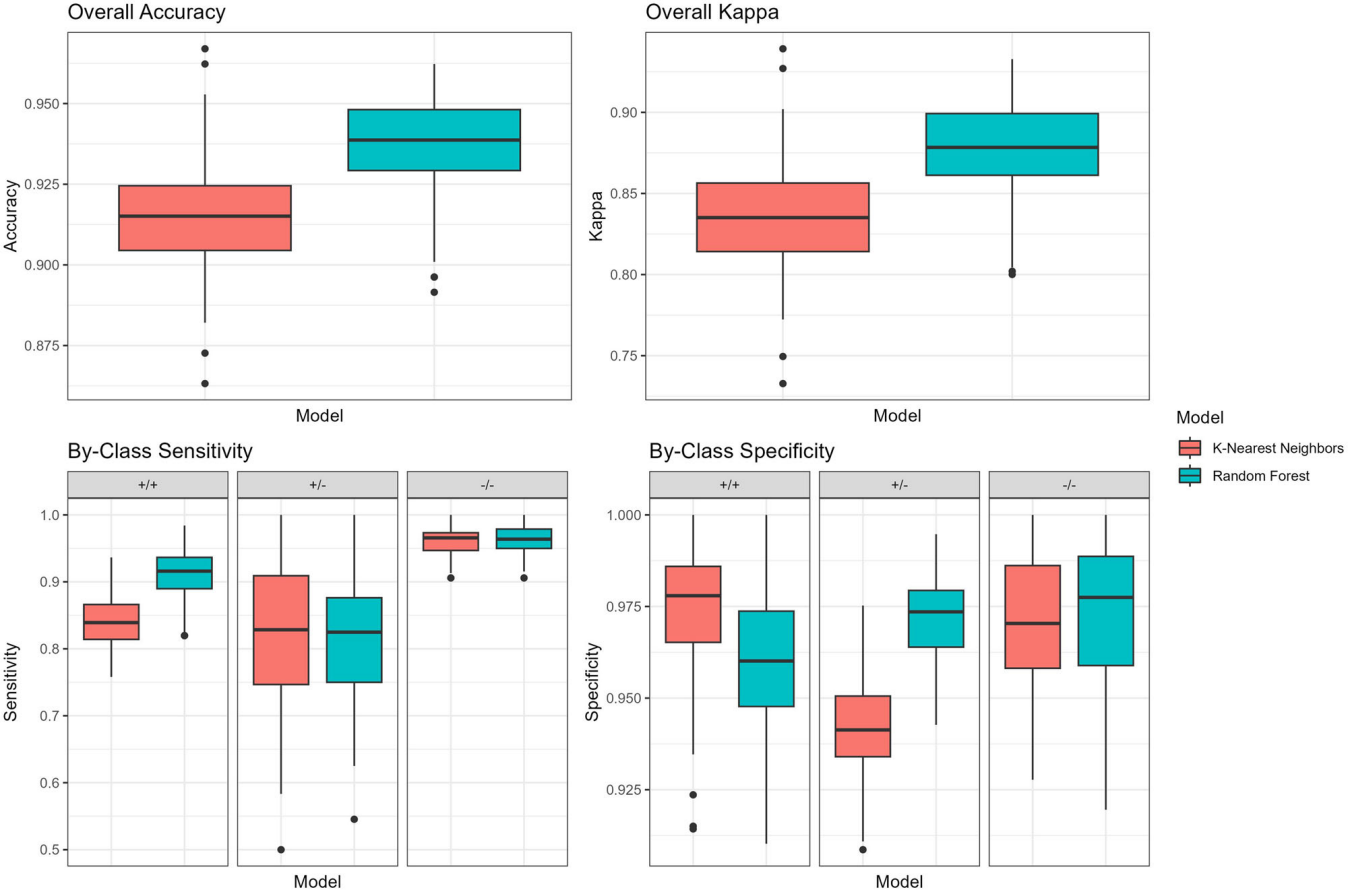
A visualization output by the “auto_locus” function of overall accuracy, kappa, and by‐class sensitivity and specificity value distributions over 100 permutations of cross‐validation. The figure legend on the right of the figure displays the color that corresponds to the model type. The top left panel displays the overall accuracy of each model in boxplots. The top right panel displays the overall kappa of each model in boxplots. The bottom left panel displays the by‐class sensitivity values in boxplots for homozygous *Sst1* individuals (+/+), heterozygous individuals (+/–), and homozygous wild‐type individuals (–/–). The bottom right panel displays the specificity of each model for each classification in boxplots. The *x* axis is the model in each figure. The *y* axis corresponds to the value of interest displayed within the graph.

Cross‐validation indicated that the training data were well suited for analysis and substantially predictive based on reported kappa values (Figure 2). Over the 100 permutations of cross‐validation, the average kappa value for the KNN model was κ=0.83 and κ=0.88 for RF. The average accuracy for the KNN model was 0.91 and 0.94 for RF. By‐class sensitivity varied by haplotype. For homozygous *Sst1* calls, KNN had a mean sensitivity of 0.84, and RF had a mean sensitivity of 0.91. For heterozygous *Sst1* calls, KNN had a mean sensitivity of 0.82, and RF had a mean sensitivity of 0.81. For homozygous wild‐type calls, both KNN and RF had a sensitivity of 0.96.

Specificities had a narrow range among haplotype classifications and models. Average specificities for homozygous *Sst1* individuals were 0.97 for KNN and 0.96 for RF. Specificities for heterozygous *Sst1* individuals were 0.94 for KNN and 0.97 for RF. Specificities for homozygous wild‐type individuals were 0.97 for both KNN and RF. These results indicate that KNN tended to under‐identify true negatives in heterozygous cases, meaning that it tended to overclassify non‐heterozygous individuals as heterozygous. Furthermore, the lower sensitivity scores of both the RF and KNN models (compared to the higher sensitivity in the homozygous cases) indicate that both models were not as well suited for classifying heterozygous individuals as they were for homozygous individuals. Based on the highest achieved average kappa value, the random forest model was selected for use in forward prediction.

**FIGURE 3:**
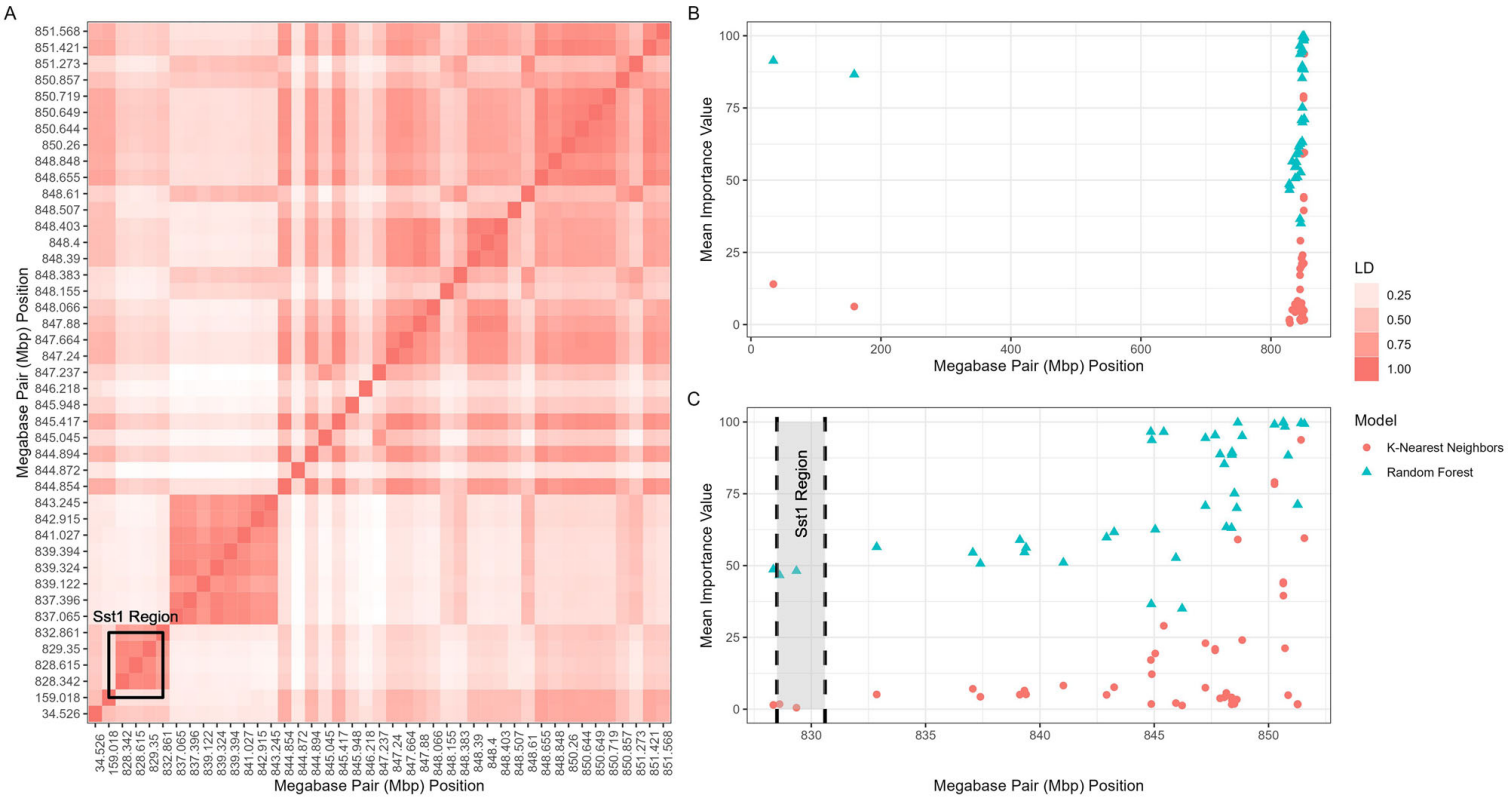
A visualization of (A) linkage disequilibrium (LD) among the most important markers identified between the k‐nearest neighbors and random forest models, (B) the importance values of markers across the genome, and (C) the importance values of markers proximal to the known position of *Sst1*. Panel (A) displays the linkage disequilibrium of each marker identified as important by the models. The *x* and *y* axes display the marker megabase pair (Mbp) position of each marker. The color within the plot on panel (A) that corresponds with the figure legend located on the right indicates the magnitude of LD between those markers. The known location of *Sst1* falls within the black box. The graph in panel (B) shows the importance of markers averaged over the 100 iterations. The *y* axis displays the importance value of the marker which is represented by the colored dot. The *x* axis represents the Mbp position of the marker. The point color corresponds with which model the point belongs to, which is denoted by the figure legend to the right. The graph in panel (C) is a zoomed in version of panel (B) where the known location of *Sst1* is labeled with a gray shaded box flanked by dashed lines.

Models in cross‐validation mainly selected markers in or near the known region of *Sst1*; however, there were two outliers on the distal short arm of 3B at ∼34 megabase pairs (Mbp) and 159 Mbp (Figure 3b). When looking at LD among the markers selected for use in the models, it appears that the outlier markers and markers in the 828–852 Mbp region share minor‐to‐substantial LD ($r^{2}:0.20<r^{2}<0.70$; Figure 3a). More specifically, the LD appears to be strong between these two outliers and markers in the 848–850 Mbp region ($\bar{r^{2}}=0.66$) which indicates that the markers may not be inherited independently. This may be the result of the misalignment of markers to the wrong arm of the 3B chromosome. Alternatively, this may be a signature of true linkage disequilibrium, indicating that some region on the short arm of 3B is being inherited frequently with the *Sst1* locus in the germplasm assessed.

When looking at derived importance values within the region, it appears that those outlier markers on the distal short arm of 3B are highly important (x>0.75) for the RF model and less so for the KNN model (x<0.25, Figure 3b). Taking a closer look at the known location of *Sst1*, it appears that markers within the region are moderately important (x=0.50) in RF models, while they were unimportant for KNN models (x<0.10) (Figure 3b). Interestingly, the most important markers (x≥0.95) identified by KNN and RF models were in the 845–853 Mbp region.

This region of highly important markers is located nearly 15–20 Mbp away from the known location of *Sst1*. However, historical markers used to haplotype the *Sst1* in the RGON and SRPN are not in perfect linkage with the causal polymorphism. Furthermore, the marker used by the Colorado State University Wheat Breeding Program, which is diagnostic of the *Sst1* locus, is found at ∼843 Mbp, which is directly adjacent to the most important markers for classification. These results may be due to the use of haplotype designations derived from markers that do not lie within or in direct proximity to the *Sst1* locus. Regardless, model performance parameters, namely kappa, indicate that both models are capable of “substantial predictions” using historical scales for kappa interpretation (Landis & Koch, 1977).

### 3.2 *Sst1* prediction forward validation

**FIGURE 4:**
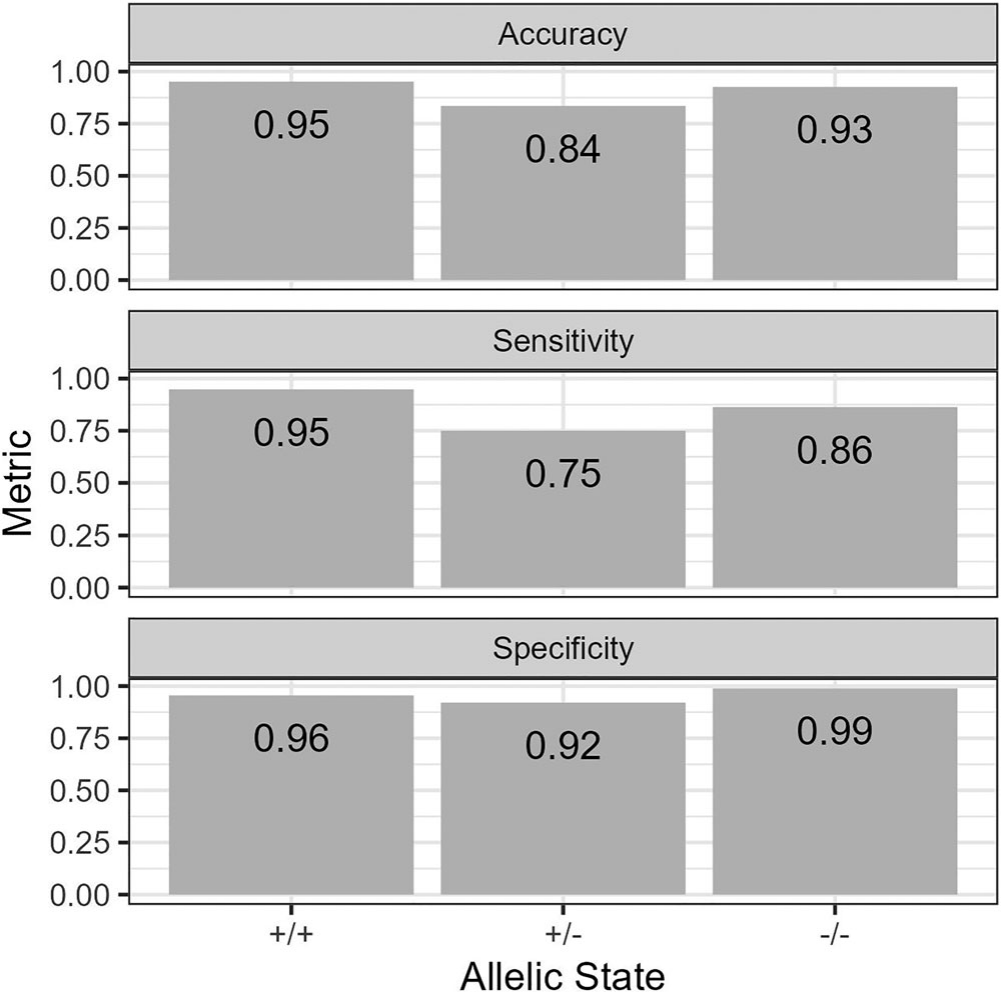
Visualization of performance parameters of predictions by a random forest model trained on all available training data. Each subgraph represents a separate measurement of model performance. The *y* axis displays the magnitude of the metric displayed in each subgraph. The allelic state on the *x* axis denotes individuals who are homozygous *Sst1* (+/+), heterozygous (+/–), and homozygous wild‐type (–/–). The value of the metric for each allelic state is displayed within each bar.

Forward validation on the WSS and AYN using an RF model trained on the total available training data produced similar results to that of cross‐validation (Figure 4). Accuracies for homozygous wild‐type and *Sst1* individuals were 0.95 and 0.93, respectively. As observed in the cross‐validation results, the accuracy for the identification of heterozygous individuals was lower at 0.84. Specificities for homozygous *Sst1*, heterozygous *Sst1*, and homozygous wild‐type were 0.96, 0.92, and 0.99, respectively. Sensitivities followed the same trend as cross‐validation, where the true positive identification rate for homozygous *Sst1* and wild‐type individuals was higher (0.95 and 0.85, respectively) than the identification of heterozygous individuals (0.75).

Based on the confusion matrix (Table 3) of predicted versus observed haplotypes, the RF algorithm misidentified heterozygous individuals as wild‐type most frequently. Homozygous wild‐type individuals were most often correctly identified (two cases misidentified), followed by homozygous *Sst1* individuals (seven cases misidentified). These results may indicate that these methods presented may be best suited for identifying homozygous individuals, like in Winn et al. (2022), rather than trying to identify heterozygous individuals as well.

**TABLE 3:** Confusion matrix of predicted *Sst1* haplotype calls versus haplotype calls made by kompetative allele‐specific polymerase chain reaction (KASP).

		KASP (observed)		
		+/+	+/−	−/−
Predicted	+/+^a^	147	7	0
	+/−^b^	6	19	15
	−/−^c^	2	0	96

^a^ Homozygous *Sst1* calls.

^b^ Heterozygous *Sst1* calls.

^c^ Homozygous wild‐type calls.

### 3.3 Estimated group means of *Sst1* haplotypes: Predicted versus genotyped

**FIGURE 5:**
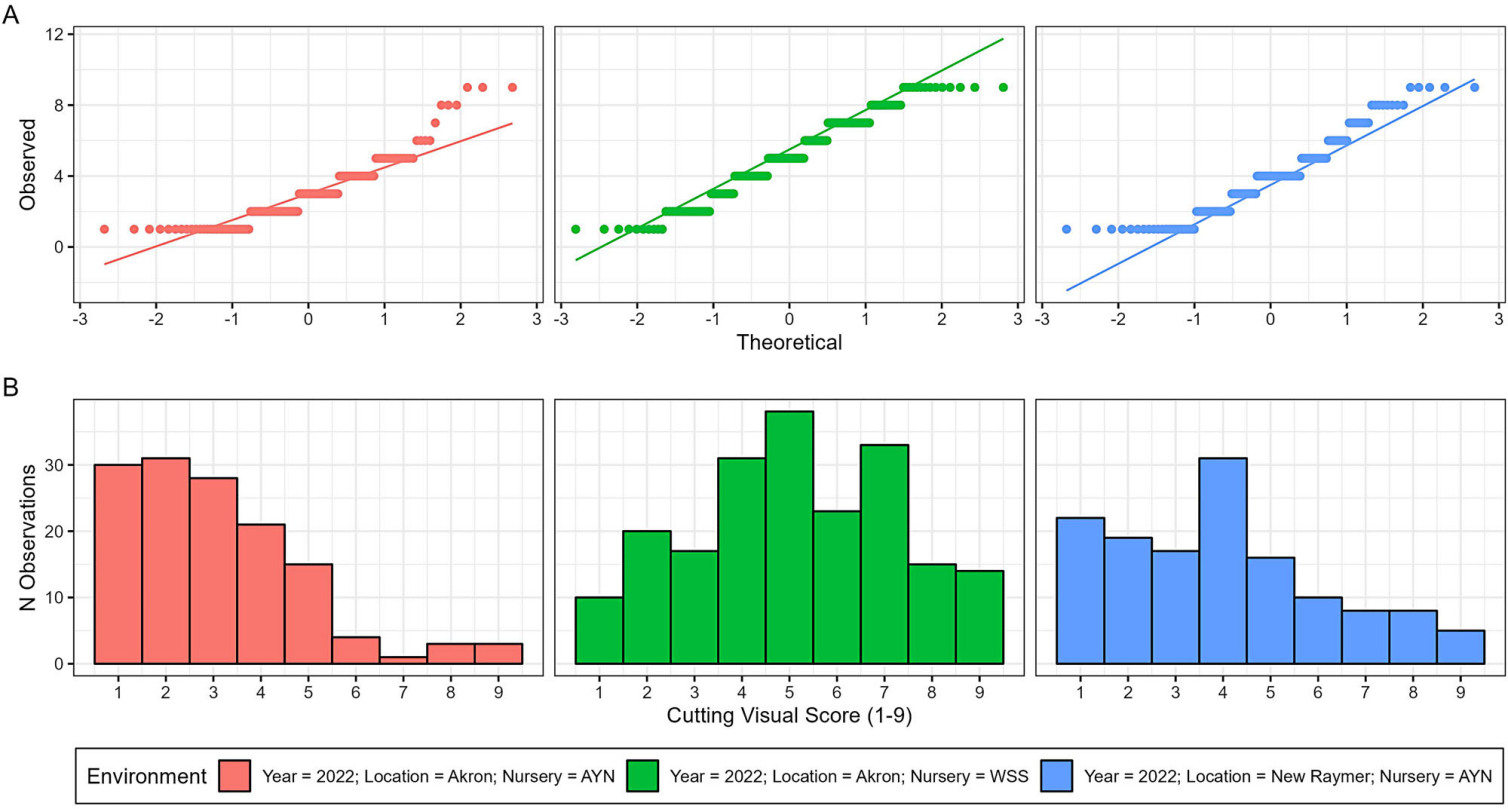
A visualization of (a) qqplots for each locations data and (b) histogram of the cutting visual score within each environment. In panel (a), the *y* axis represents the observed cutting visual score, and the *x* axis represents the theoretical quantiles. The line going across observation points shows the pattern of expected versus observed visual scores for a normal distribution. Panel (b) displays histograms of each location where the *y* axis is the count of observations within the bin, and the *x* axis is the cutting visual score. The legend at the bottom of the image displays each environment which corresponds to the color of each subgraph. AYN, advanced yield nursery; WSS, wheat stem sawfly solid stem panel.

Visual examination of distributions for both locations of the AYN revealed somewhat skewed data distributions, while observations from the single location of the WSS followed an approximately normal distribution (Figure 5). Notably, the AYN exhibited a distribution skewed toward lower values of cutting visual scores at both field sites in Akron, Colorado, and New Raymer, Colorado. This is most likely because the AYN is one generation later than the WSS in the breeding process and has already gone through one cycle of selection for wheat stem sawfly resistance. Summary statistics of the location mean, minimum, maximum, standard deviation, heritability, and standard error of heritability are also provided (Table 4).

**TABLE 4:** Table of descriptive statistics for each environment.

Year	Location	Nursery	N observations	N genotypes	Min	Mean	Max	SD^a^	*h* ^2^ *_g_* ^b^	SE^c^
2022	Akron	AYN	136	107	1.00	3.07	9.00	1.86	0.62	0.10
2022	New Raymer	AYN	136	107	1.00	3.97	9.00	2.23	0.70	0.08
2022	Akron	WSS	201	185	1.00	5.12	9.00	2.18	0.49	0.11

Abbreviations: AYN, advanced yield nursery; Max, maximum; Min, minimum; WSS, wheat stem sawfly solid stem panel.

^a^ Standard deviation of cutting visual score.

^b^ Narrow‐sense, per‐plot, genomic heritability.

^c^ Standard error of heritability measurements.

Narrow‐sense, per‐plot, genomic heritabilities varied among locations. The lowest heritability ($h_{g}^{2}=\;0.49\pm 0.11$) was observed in Akron, Colorado, for the WSS, and the highest ($h_{g}^{2}=\;0.70\pm 0.08$) was observed in New Raymer, Colorado, for the AYN. The mean cutting score in both Akron and New Raymer was lower for the AYN ($\bar{x}=\;3.07$ and $\bar{x}=\;3.97$, respectively) than Akron for the WSS ($\bar{x}=\;5.12$); however, both nurseries across locations contained visual scores between one and nine. This implies that the generation of selection prior to the AYN did shift the population mean toward resistance, yet it did not cull out all susceptible genotypes, which is expected.

**FIGURE 6:**
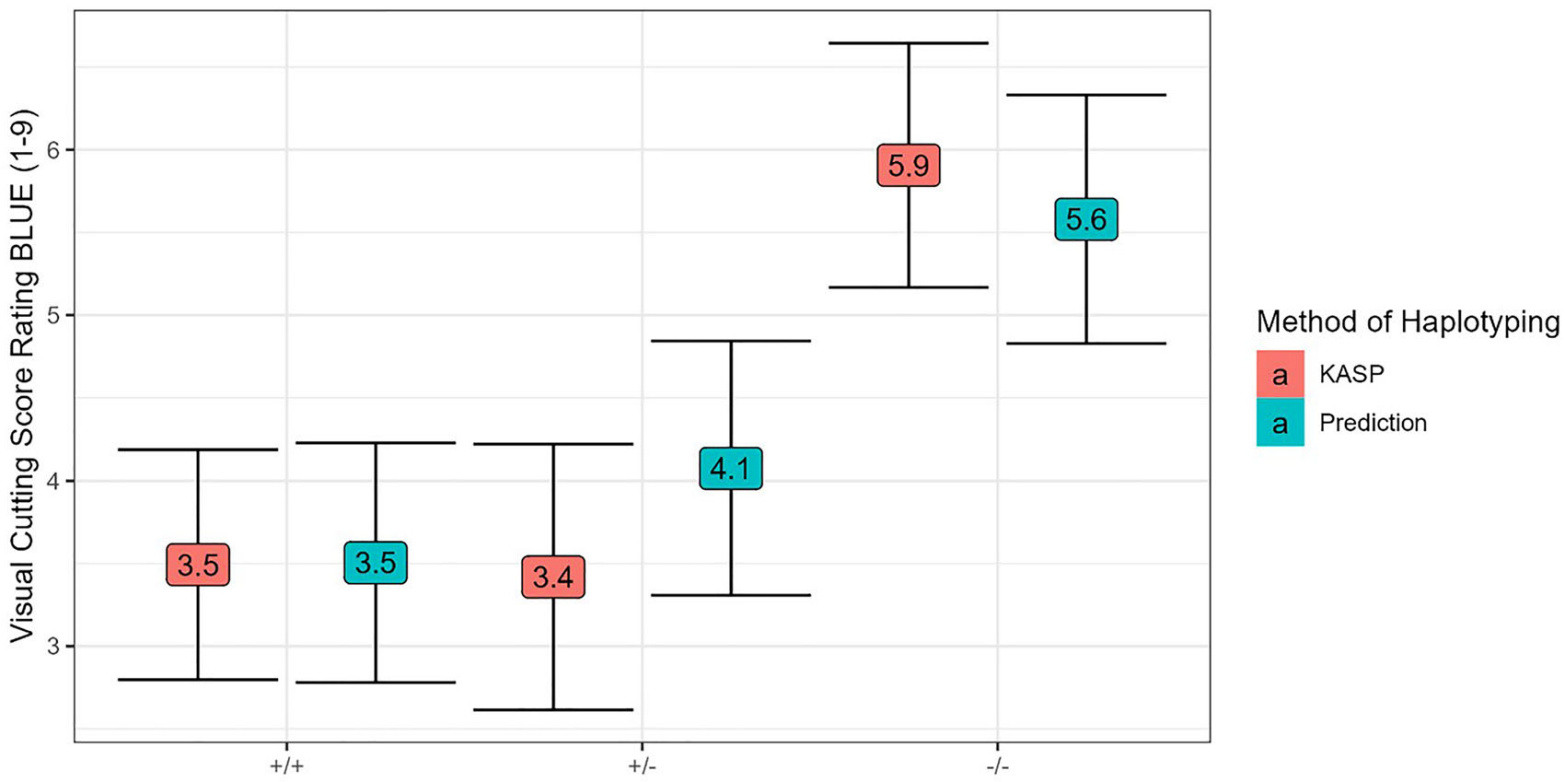
Kompetative allele‐specific polymerase chain reaction (KASP)‐derived *Sst1* haplotype call effects versus predicted *Sst1* haplotype effects. On the *x* axis is the allelic state of the *Sst1* locus where +/+ represents individuals homozygous for *Sst1*, +/− represents individuals as heterozygous for the *Sst1* locus, and −/− represents individuals homozygous for the wild‐type allele at the *Sst1* locus. The *y* axis displays the visual cutting score rating best linear unbiased estimate (BLUE) for the estimated effect of *Sst1*. The legend on the right indicates what color‐coded box corresponds to which type of *Sst1* haplotype assignment. The black bars around each point estimate represents a 95% confidence interval about the estimate.

Both predicted and KASP‐genotyped *Sst1* haplotype calls had significant effects on cutting scores (P(F)<0.05). Homozygous *Sst1* and heterozygous *Sst1* individuals did not have substantially different cutting scores when classifying based on either KASP‐genotyped or predicted *Sst1* haplotypes. Estimates of *Sst1* effects made by prediction were not significantly different from KASP‐genotyped *Sst1* effects within each haplotype (Figure 6). In the case of KASP‐genotyped *Sst1* effects, the homozygous wild‐type individuals significantly differentiated themselves from both the homozygous *Sst1* and heterozygous individuals; however, predicted haplotypes for homozygous wild‐type individuals did not significantly differentiate from heterozygous individuals. This is because the prediction method tended to incorrectly classify homozygous wild‐type individuals as heterozygous (Table 3).

## 4 DISCUSSION

In the current study, we developed an R user‐accessible R package by the name “HaploCatcher” which can predict haplotypes using historical information derived from molecular marker assays on genome‐wide genotyped lines. The function, “auto_locus,” allows users to produce predictions for the many lines submitted for sequencing which are not KASP genotyped on an annual basis. Just as in the work performed by Winn et al. (2022), we suggest that this method may be deployed in generations where genome‐wide sequencing is performed on a very large number of lines which would otherwise not be screened via polymerase chain reaction (PCR)‐based assays for these loci. While these predictions were not perfect in their predictive ability (k=1), they were substantial (k≥0.80) and similar with respect to the results of Winn et al. (2022) (Landis & Koch, 1977). Here, we demonstrated that this method is successful in predicting the *Sst1* locus which has a direct impact on improving sawfly resistance in areas threatened by this emerging pest. Moreover, applying this method to resistance loci beyond *Sst1* could lead to further progress in the pyramiding and maintenance of wheat stem sawfly resistance.

The cross‐validation results in the current work were similar to those in Winn et al. (2022); however, unlike Winn et al. (2022), we included the option “include_hets” in the “auto_locus” function arguments, which allows for the prediction of biallelic loci with a heterozygous state. In our results, we observed that both the KNN and RF models were not as capable of identifying heterozygous cases as they were homozygous cases. There may be several reasons for this phenomenon.

First, lines in the Colorado State University Wheat Breeding Program are often initially sequenced in the F_3:5_ generation with recurrent sequencing in each subsequent year. Leaf tissue from 10 seeds of each line is bulked and used to prepare libraries for sequencing. Therefore, if a line that is heterozygous for the *Sst1* locus was selected in the F_3_ generation, the bulked DNA extracted in the F_3:5_ would be heterogenous at the *Sst1* locus, and because of this, the SNPs detected in the *Sst1* region may not be truly representative of a heterozygous *Sst1* haplotype, leading to misidentification by this method.

Second, we curated KASP data produced by the USDA Central Small Grains genotyping laboratory over years for training. These data, while highly informative, showed some inconsistency across the years. Marker platforms, haplotyping call formats, and locus region sizes change over the years, and this can lead to unexpected associations of markers with the locus. More specifically, we observed that markers 15–20 Mbps away from the known region of the locus were identified as “highly important.” This may be because markers that were used to haplotype the region were not in direct linkage with the causal polymorphism, and this led to the detection of markers on the distal long arm of 3B as important.

Furthermore, we aligned our genetic data to the IWGSC wheat reference genome version 2.0 (Appels et al., 2018); this genome is genetically distant from the wheat germplasm located in the Great Plains area of the United States and may have led to misalignments of sequencing reads, like those potentially observed in the marker importance figure (Figure 3a). Moreover, these genetic data were imputed using the Beagle algorithm (Browning et al., 2018), which is not perfectly predictive. Therefore, the summed errors of the genomic sequencing method, historical data curation, misalignment, and imputation may have contributed to the lower predictability of heterozygous classes. We then suggest that if users can do so, they produce their own training populations within their own programs and genotype them with a consistent set of markers for the best results possible. Regardless, our proposed models are still substantially informative.

When comparing KASP‐based and predicted *Sst1* haplotype call group mean estimates, we observed that the predicted and KASP‐based haplotype group means were not significantly different from each other within the haplotype. We did observe that homozygous wild‐type and heterozygous *Sst1* haplotype group means did not significantly differentiate in the prediction as the RF model used to make this prediction often grouped heterozygous individuals with wild‐type haplotypes (Table 3).

Irrespective of these shortcomings, this method provides a way to assess haplotypes of interest, with a measurable margin of error, in generations that would otherwise not be screened. This method, as shown in the work by Winn et al. (2022), can be generalized to loci which affect many different traits. The potential application of this method could be broadened to traits other than disease resistance as well, so long as programs have a backlog of lines with informative haplotype and genome‐wide marker data. More importantly, this package, “HaploCatcher,” now provides an easily accessible method of pipeline implementation for breeding programs.

While targeted sequencing platforms (Lundberg et al., 2013) may reduce the need for a method like this, the method utilized in the “HaploCatcher” package will remain useful for programs without access to targeted sequencing platforms or programs missing probes for specific loci of interest. Furthermore, this method can accommodate many different sequencing platforms (diversity arrays, genotyping‐by‐sequencing, amplicon, etc.), does not require physical position information, and can potentially be widely applied across species. Lastly, we demonstrated that this method can be applied within breeding programs and produce comparable results to PCR‐based marker calls; more specifically, we showed that this method could be a viable way of screening early development germplasm for the *Sst1* locus, and thus increase the frequency of this locus earlier in the development pipeline.

## 5 CONCLUSION

The utility of marker‐assisted selection has been vetted through the vast literature available for the method. However, with whole genome sequencing technologies being applied in early generations for use in genomic prediction, there lies an opportunity to acquire data on haplotypes of important loci. The method proposed in Winn et al. (2022) allows breeding programs to organize their historical marker‐assisted selection data to produce predictive haplotype calls for lines in generations where PCR‐based assays for loci of interest are not run due to increased time, labor, and genotyping cost. This can allow breeders to observe haplotype profiles of potential varieties much earlier in the breeding process than before. Here, we chose wheat stem sawfly—an emerging pest that threatens grower profitability and the dryland cropping agroecosystem—as a test case to demonstrate the effectiveness of this method. We used existing genotypic datasets to deliver breeders precise predictions of the presence of a major resistance gene, *Sst1*, allowing for improved selection for stem sawfly resistance at an earlier generation. With the development of the “HaploCatcher” package, there is now freely accessible software for easier implementation of this method in other breeding pipelines.

## AUTHOR CONTRIBUTIONS

**Zachary James Winn**: Conceptualization; data curation; formal analysis; investigation; methodology; project administration; resources; software; supervision; validation; visualization; writing‐original draft; writing‐review and editing. **Emily Hudson‐Arns**: Data curation; writing‐original draft; writing‐review and editing. **Mikayla Hammers**: Investigation; methodology; software; writing‐review and editing. **Noah DeWitt**: Investigation; methodology; writing‐original draft; writing‐review and editing. **Jeanette Lyerly**: Conceptualization; investigation; methodology; software; validation; writing‐review and editing. **Guihua bai**: Data curation; resources. **Paul St. Amand**: Data curation; resources; writing‐review and editing. **Punya Nachappa**: Data curation; writing‐review and editing. **Scott Haley**: Data curation; funding acquisition; resources; writing‐review and editing. **Richard Esten Mason**: Conceptualization; funding acquisition; investigation; resources; writing‐review and editing.

## CONFLICT OF INTEREST STATEMENT

The authors declare no conflicts of interest.

## DATA AVAILABLILITY STATEMENT

Code and data utilized in this study may be found at https://github.com/zjwinn/HAPLOCATCHER‐AN‐R‐PACKAGE‐FOR‐PREDICTION‐OF‐HAPLOTYPES. The package developed for this project, “HaploCatcher,” can be directly downloaded to an R installation using devtools::install_github(“zjwinn/HaploCatcher”) to directly install from GitHub or install.packages(“HaploCatcher”) to install from the CRAN database. Questions related to data input formatting or function arguments may be found in the relevant help files available in the package description within R statistical software installations.

## Supporting information

Supplementary Information
