# Supplementary material for: HaploCatcher: An R package for prediction of haplotypes
Source: Plant Genome. 2023 Nov 15;17(1):e20412. doi: 10.1002/tpg2.20412 (PMC12807199; doi:10.1002/tpg2.20412)
Supplement: Supplementary file 1 [file TPG2-17-e20412-s001.docx]

# Supplementary Information

Supplementary Information 1. R code to access vignettes for information on HaploCatcher inputs written as an R markdown chunk. To access these relevant help files and vignettes, users must install the HaploCatcher package and call upon relevant help files using the code below.

```{r}

install.packages(“HaploCatcher”)

library(HaploCatcher)

vignette("An_Intro_to_HaploCatcher", package="HaploCatcher")

help("auto_locus", package = "HaploCatcher")

help("geno_mat", package = "HaploCatcher")

help("gene_comp", package = "HaploCatcher")

help("marker_info", package = "HaploCatcher")

```
